# Comparative Study of Continuous-Flow Reactors for Emulsion Polymerization

**DOI:** 10.3390/polym17172289

**Published:** 2025-08-24

**Authors:** Kai-Yen Chin, Angus Shiue, Pei-Yu Lai, Chien-Chen Chu, Shu-Mei Chang, Graham Leggett

**Affiliations:** 1 Department of Molecular Science and Engineering, National Taipei University of Technology, Taipei 10608, Taiwan; f104gstyle@gmail.com (K.-Y.C.); angusshiue@gmail.com (A.S.); skittylie890805@gmail.com (P.-Y.L.); mala19970502@gmail.com (C.-C.C.); 2 LI-COR, Lincoln, NE 68504, USA; graham.leggett@licor.co

**Keywords:** styrene, microreactor, mini-emulsion polymerization

## Abstract

Polymer fouling in batch and tubular reactors creates safety hazards from heat buildup and blockages. The continuous Corning Advanced-Flow™ Reactor (AFR) offers enhanced mass and heat transfer, improving safety and efficiency. This study evaluated three reactor systems—a monolithic AFR, an AFR with an external pipe, and a conventional tubular reactor—for the mini-emulsion polymerization of styrene and subsequent styrene–acrylic acid copolymerization. The AFR operability under varying monomer concentrations was assessed and investigated, with the residence time’s effects on conversion. For styrene polymerization at 20–35 wt% monomer, the highest conversions achieved were 88.0% in the AFR, 85.8% in the tubular reactor, and 98.9% in the AFR with pipe. Uniform particles were obtained at ≤30 wt%, whereas at 35 wt%, the monolithic AFR experienced clogging and loss of particle uniformity. Similarly, in styrene–acrylic acid copolymerization (15–17.5 wt% monomer), the maximum conversions reached 80.1% in the AFR and 95.4% in the AFR with pipe, while the monolithic AFR again experienced blockage at 17.5 wt%. In conclusion, integrating an external pipe with the AFR, coupled with higher flow rates, significantly improved initiator diffusion, enhanced monomer conversion, and mitigated blockage. This approach enabled the efficient, continuous production of nanoscale, uniformly sized polystyrene and styrene–acrylic acid copolymers even at high monomer concentrations.

## 1. Introduction

Emulsion polymerization is a foundational technique for synthesizing polymers used in a diverse range of applications, such as adhesives, paints, paper additives, textiles, plastic composites, and construction materials [1,2,3]. For instance, it is instrumental in the production of engineering plastics such as acrylonitrile–butadiene–styrene (ABS) and styrene–butadiene rubber (SBR), which exhibit excellent physical and chemical properties suited to a wide range of applications [4,5,6]. However, a major challenge in large-scale industrial emulsion polymerization—typically conducted in batch or semi-batch tank reactors—is the persistent problem of fouling deposit formation [7].

These deposits can cause blockages and force unplanned operational shutdowns, thereby undermining production efficiency. Although motor-driven impellers and internal baffles are engineered to enhance flow patterns and mixing performance in such reactors, they often encounter limitations such as insufficient mixing and pronounced temperature gradients between the reactor core and its periphery. Moreover, depending on the specific configuration of the agitator, completely stagnant “dead zones” may develop within the tank, further compromising reaction uniformity and heat transfer [7,8,9].

The presence of dead zones—areas with sluggish flow and extended local residence times—undermines overall mass transfer efficiency. This results in lower local reactant concentrations, leading to non-uniform polymer molecular weight distribution, inconsistent polymer latex particle sizes, decreased suspension stability, and eventual gravitational settling due to particle agglomeration. This undesirable deposition of polymer latex, known as polymerization fouling, can adhere to or adsorb onto internal surfaces such as reactor walls and mixing elements. A critical consequence is the formation of fouling layers on heat transfer surfaces, which significantly increases thermal resistance and reduces heat transfer efficiency. Due to their inherently low thermal conductivity (λ = 0.1–0.5 W·m^−1^·K^−1^), even polymer deposits just a few micrometers thick can significantly hinder heat transfer [10,11].

Continuous-flow microreactor technology is regarded as an attractive solution for process miniaturization [12,13]. Continuous tubular reactors, in particular, offer higher productivity by leveraging a greater surface area-to-volume ratio, which enhances heat dissipation and supports higher polymerization rates without triggering runaway reactions [14,15,16,17,18]. This, in turn, reduces the risk of producing large volumes of off-spec material requiring disposal [14,19].

However, a significant limitation of continuous-flow microreactors is their scalability, which prevents them from being a complete replacement for existing systems in large-scale polymer production. Although the viability of continuous tubular microreactors for polymer latex production via emulsion polymerization has been previously demonstrated [20,21,22], these reactors are particularly prone to solid accumulation (fouling) on their inner walls. This can eventually lead to clogging and complete reactor failure [23,24]. Until now, such polymerization processes have primarily employed tubular reactors lacking integral mixing capabilities. Recent assessments have investigated alternative systems, including Corning’s Advanced-Flow™ glass reactors (AFRs), which incorporate a continuous heart-shaped design [25,26,27]. These reactors utilize millimeter-scale channel modules equipped with integrated micromixer geometries engineered to enhance thermal management and interfacial mass transfer, thereby minimizing overall heat and mass transfer resistances [28,29]. However, this type of commercial system has not yet been extensively studied for emulsion polymerization, especially in terms of conversion efficiency, operational stability, pressure drop, and detailed analysis of polymerization fouling through imaging [27,30].

The objective of this study was to evaluate the practical applicability of three continuous-flow reactors—a custom-built tubular reactor, the Corning Advanced-Flow Reactor (AFR), and the Corning AFR integrated with an external PTFE pipe—in emulsion polymerization processes. The comparison was carried out by synthesizing polystyrene and styrene–acrylic acid copolymers under comparable conditions. To prevent particle size-dependent colloidal instability, pre-emulsions were uniformly emulsified to droplet sizes below 100 nm using an ultrasonic method. The study meticulously monitored monomer conversion, operational stability, pressure drop, and internal reactor conditions throughout the polymerization process.

## 2. Materials and Methods

### 2.1. Materials

Styrene (St, ≥99%, Sigma, St. Louis, MO, USA) and acrylic acid (AA, ≥99%, First Chem, Taipei, Taiwan) were used as monomers. Prior to use, all monomers were purified by passing through basic alumina columns to remove inhibitors. The following reagents were used as received without further purification: sodium dodecyl sulfate (SDS, SHOWA, Tokyo, Japan, ≥90%), n-hexadecane (HD, Alfa Aesar, Heysham, UK, ≥99%), potassium persulfate (KPS, ACROS, Sint-Niklaas, Belgium, ≥99%), sodium bicarbonate (NaHCO3, ACROS, Sint-Niklaas, Belgium, 99.5%), methanol (Baker, KR, ≥99%), and tetrahydrofuran (THF, Duksan, KR, ≥99%). Deionized water prepared in our laboratory was used throughout the experiments.

### 2.2. Experimental Setup

**Figure 1:**
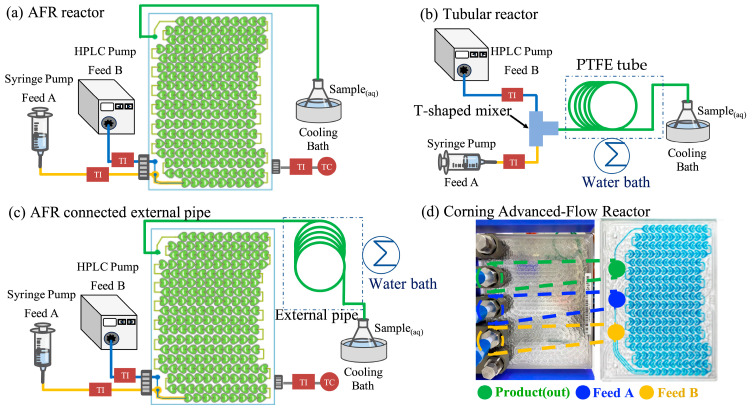
Schematic diagram of the experimental setup: (**a**) Corning Advanced-Flow Reactor (AFR), (**b**) tubular reactor, and (**c**) AFR connected to an external pipe to extend residence time. (**d**) Structural layout of the Corning AFR, featuring its multilayer glass design for efficient heat exchange and continuous-flow processing.

The schematic diagram of the experimental setup is shown in Figure 1, which includes three types of reactors: (a) a Corning Advanced-Flow Reactor (AFR, Figure 1a), (b) a tubular reactor (Figure 1b), and (c) a Corning AFR connected to an external PTFE pipe (Figure 1c). The Corning AFR (Figure 1d) is constructed from glass and comprises three layers. The outer layer circulates silicone oil for temperature control, while the middle layer contains the internal mixing section featuring a heart-shaped microstructure with 200 mixing units, providing a total internal volume of 2.7 mL. Fluids are pumped through the reactor, where efficient mixing is achieved via continuous splitting, recombination, and directional flow changes within the microstructure.

The tube reactor comprises a T-shaped mixer connected to a PTFE tube (inner diameter: 1.6 mm), which is heated using a constant-temperature water bath. However, at high feed rates, the limited internal volume of the AFR may result in insufficient residence time. To address the limitation in residence time, a PTFE tube with the same inner diameter as the tube reactor was connected to the outlet of the AFR, effectively extending the overall flow path. This additional tubing was immersed in a constant-temperature water bath to maintain consistent thermal conditions throughout the reaction. All polymerization reactions were conducted at a controlled temperature of 80 °C.

Reactants were introduced separately into the reactors using a syringe pump and an HPLC pump. Feed Tank A preparation: A monomer emulsion was prepared by ultrasonicating a mixture containing 40 wt% monomer (either styrene (St) or a styrene–acrylic acid (St + AA) blend) and 1 wt% HD in an aqueous solution of SDS at concentrations of 0.5, 1, 2, 3, or 4 wt%. Feed Tank B contained an aqueous solution prepared in a vial, consisting of 2.5 wbm% KPS and sodium bicarbonate (NaHCO_3_). The emulsion was ultrasonicated for 30 min at 21% amplitude (corresponding to 21–25 W) using a Q700 sonicator (Qsonica, India) equipped with a 0.5-inch diameter probe. To avoid heat-induced polymerization during monomer ultrasonication, the ultrasonicator was pulsed on and off at intervals, while the emulsion was continuously cooled in an ice bath throughout homogenization.

The flow rate ratio between the initiator solution and the monomer emulsion was adjusted to achieve final concentrations of 1 wt% initiator and 20, 30, or 35 wt% monomer for styrene polymerization. For the styrene–acrylic acid (St + AA) copolymerization, the final monomer concentrations were adjusted to 10, 15, and 17.5 wt%, with the initiator concentration maintained at 1 wt%. The detailed feed formulations and corresponding flow rates introduced into the reactor are summarized in Table S1. Samples were collected at the reactor outlet, excluding the first three to ensure steady-state conditions. Immediately after collection, 1 wt% hydroquinone solution was added to each sample to quench the reaction prior to further analysis.

### 2.3. Characterization

The polydispersity index, average particle size, and droplet size of the latexes and emulsions were analyzed using dynamic light scattering (DLS, Nano Brook 90 Plus, Brookhaven Instruments Corporation, Holtsville, NY, USA). Prior to measurement, all latex and emulsion samples were diluted 250-fold with an 8.2 mM SDS solution.

Field emission scanning electron microscopy (FE-SEM) analysis was performed using a Hitachi Regulus 8100 microscope operated at an accelerating voltage of 3 kV. Diluted latex samples were deposited onto 1 × 1 cm silicon (Si) wafers and allowed to air dry. The dried samples were subsequently coated with a thin conductive layer of gold (Au) by sputter coating prior to imaging. Monomer conversion was determined gravimetrically.

The resulting product was separated by centrifugation at 9000 rpm, and the solid residue was repeatedly washed with methanol. Gel permeation chromatography (GPC) was used to determine the molecular weights and molecular weight dispersity (Đ, Mw/Mn) of the polymers. The system was equipped with a refractive index detector (RID-20A, Shimadzu, Kyoto, Japan) and TSKgel columns (Shimadzu, Kyoto, Japan). Tetrahydrofuran (THF) was used as the eluent at a flow rate of 1.0 mL/min. The calibration curve was constructed using polystyrene standards.

## 3. Results and Discussion

### 3.1. Optimization of Monomer Emulsification and Emulsion Stability

To achieve colloidally stable emulsions, minimizing the final emulsion droplet size for a given amount of stabilizer is essential. Therefore, a series of ultrasonic emulsification experiments were conducted to identify the optimal surfactant concentration needed to stabilize the smallest possible droplet sizes. The effect of varying sodium dodecyl sulfate (SDS) concentrations on the emulsion droplet size was systematically investigated. SDS concentrations of 0.5, 1, 2, 3, and 4 wt% (relative to the total emulsion) were evaluated with ultrasonication times ranging from 1 to 30 min. Emulsion droplet sizes were measured within one hour after ultrasonication to assess the immediate stability and size distribution following homogenization.

**Figure 2:**
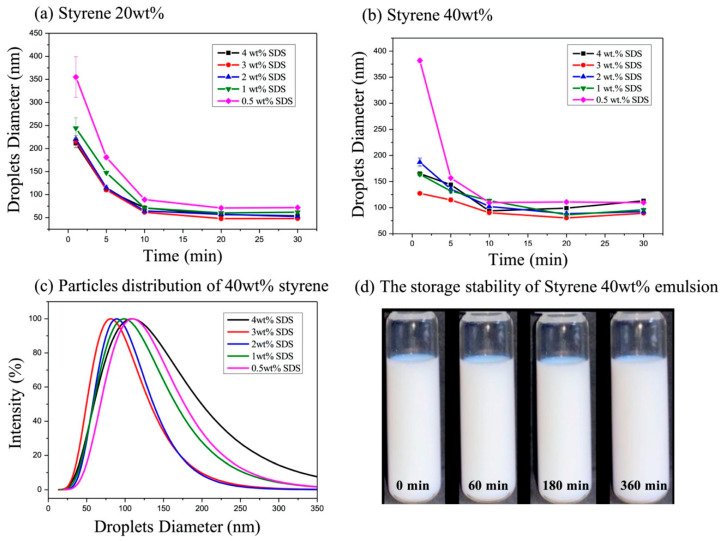
Effect of surfactant concentration on emulsion particle size and stability: (**a**) average droplet size of emulsions containing 20 wt% styrene at varying SDS concentrations; (**b**) average droplet size of emulsions containing 40 wt% styrene under the same conditions; (**c**) droplet size distribution of the emulsions at different SDS concentration; (**d**) stability evaluation of the styrene (St) emulsion at 3 wt% SDS over six hours.

As shown in Figure 2a,b, increasing the SDS concentration resulted in a reduction in the surface tension of the oil droplets, leading to smaller droplet sizes [31]. Figure 2c further demonstrates that a surfactant concentration of 3 wt% with 20 min of ultrasonication produced the smallest styrene (St) emulsion droplet size, measuring 80.3 ± 1 nm, with a size distribution (PDI = 0.156). However, increasing the SDS concentration to 4 wt% for 20 to 30 min of high-energy ultrasonic emulsification led to an increase in the droplet size, to 100.3 ± 5 nm and 123.4 ± 13 nm, likely due to re-coalescence [31,32,33]. Additionally, the droplet size distribution broadened significantly, as indicated by a PDI of 0.302. To prevent agglomeration, 3 wt% SDS was selected as the optimal surfactant concentration, and an ultrasonic homogenization time of 20 min was identified as providing the most effective emulsification conditions. The stability of the St emulsion was subsequently evaluated, as shown in Figure 2d. The optimized emulsion—prepared using 3 wt% SDS and subjected to 20 min of ultrasonic treatment—exhibited no observable phase separation even after six hours.

**Figure 3:**
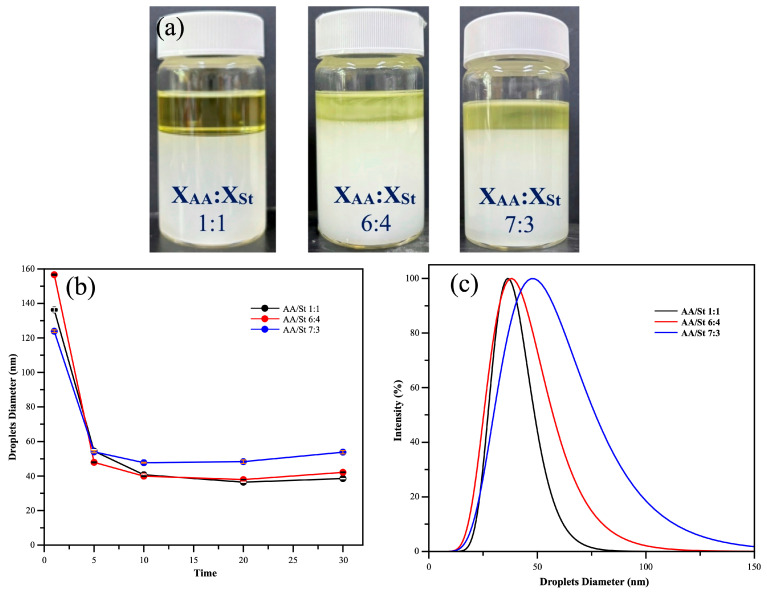
(**a**) The stability of St/AA emulsion when the total monomer content is 40 wt%. Significant phase separation occurs within 10 min. (**b**) Effect of ultrasonic emulsification time on droplet size at a total monomer content of 20 wt%. (**c**) Droplet size distribution of St/AA emulsion with different molar ratios.

The emulsification conditions optimized for the 40 wt% styrene emulsion were not suitable for the 40 wt% styrene–acrylic acid (St/AA) co-emulsion. Figure 3a presents photographs of the 40 wt% St/AA co-emulsions at various molar ratios (1:1, 6:4, and 7:3). These co-emulsions exhibited significant phase separation, with more than 20% breakup observed. In contrast, the 20 wt% St/AA co-emulsion was successfully emulsified. The emulsification conditions and resulting droplet size distribution for this concentration are shown in Figure 3b,c. The smallest droplet size of 36.52 nm was achieved using a 1:1 molar ratio of St to AA with 20 min of homogenization.

As the molar ratio of acrylic acid (AA) increases, the emulsion droplet size tends to increase. This trend can be attributed to the polar and hydrophilic nature of the carboxylic groups in acrylic acid, which influence the emulsion’s stability and droplet formation. In an aqueous environment, the carboxylic acid groups of acrylic acid (AA) may not be fully protonated, especially under neutral or low-pH conditions [34]. As a result, AA may not effectively contribute to electrostatic repulsion, limiting its ability to function as a co-surfactant. Additionally, the hydrophilic character of acrylic acid (AA) may lower the interfacial free energy, which can actually lead to decreased emulsion stability. In other words, increasing the AA content makes it harder to form a stable, concentrated emulsion [35,36].

Furthermore, the hydrophilic nature of AA may reduce the interfacial free energy, which can compromise emulsion stability. Simply put, increasing the AA content makes it more difficult to form a stable concentrated emulsion.

### 3.2. Practical Applicability of the Three Types of Continuous-Flow Reactors

In the previous section, optimal emulsions were prepared and subsequently used to evaluate the practical applicability of three types of continuous-flow reactors. By adjusting the flow rate ratio of Feed A and Feed B, the monomer concentrations entering the reactors for polymerization were controlled at 20 wt%, 30 wt%, and 35 wt% for the styrene (St) emulsion, and at 10 wt%, 15 wt%, and 17.5 wt% for the styrene–acrylic acid (St/AA) co-emulsion. To maintain a fixed initiator-to-monomer ratio of 1 wt% during polymerization in the AFR, initiator aqueous solutions with different concentrations must be prepared to correspond with the varying monomer concentrations.

#### 3.2.1. AFR Operability Analysis

For the Advanced-Flow Reactor (AFR) system, the internal volume is fixed at 2.7 mL. Given the pump’s minimum total flow rate limit of approximately 0.2 mL/min, the maximum achievable residence time is around 14 min. Detailed operating conditions are provided in Table S2.

##### Emulsion Polymerization of Styrene

**Figure 4:**
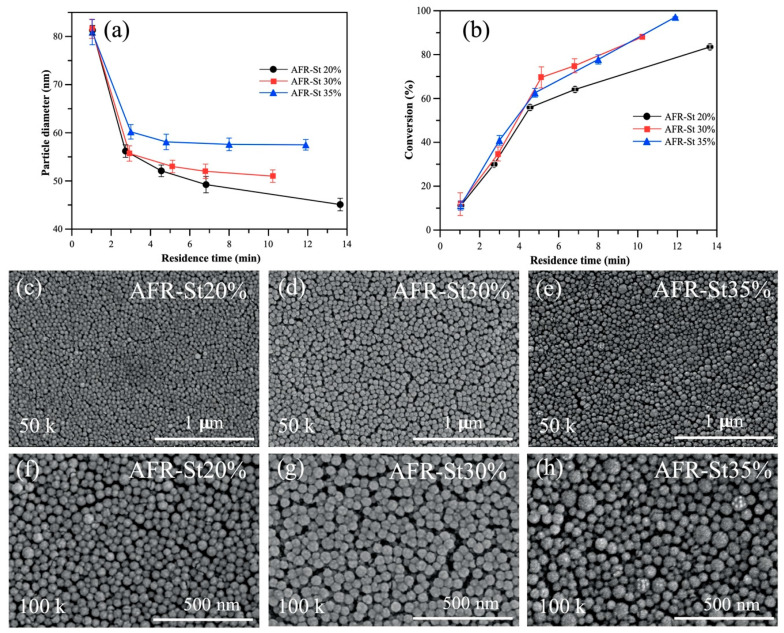
Effect of styrene (St) monomer concentration on (**a**) latex particle size and (**b**) conversion. Scanning electron microscopy (SEM) images of polystyrene spheres: (**c**,**f**) AFR-St20%, (**d**,**g**) AFR-St30%, and (**e**,**h**) AFR-St35%.

Figure 4a illustrates the results of styrene (St) emulsion polymerization. At similarly short residence times, variations in the monomer concentration had minimal influence on conversion during the initial stages of polymerization. However, when the residence time exceeded 8 min, conversion increased with higher monomer concentrations.

At a monomer concentration of 35 wt%, the highest conversion of 97 ± 0.2% was achieved at a residence time of 12 min, producing a polymer with Mn = 260 kDa, Mw = 431 kDa, and Đ = 1.66. For a concentration of 30 wt%, conversion reached 88 ± 0.4% at 10 min residence time (Mn = 403 kDa, Mw = 632 kDa, Đ = 1.66). At a lower concentration of 20 wt%, a conversion of 84 ± 1% was obtained after 14 min, with Mn = 283 kDa, Mw = 450 kDa, and Đ = 1.59.

##### Latex Particle Size and Morphology

Figure 4b illustrates the pronounced influence of the residence time on the particle size of the resulting styrene latex. At a residence time of approximately 3 min, the particle size showed a reduction of less than 25%. With further increases in the residence time, the particle size progressively decreased. The smallest particles, measuring 45.1 ± 1 nm, were obtained at a 14-minute residence time with a 20 wt% monomer concentration. In comparison, particle sizes of 52.1 ± 1 nm and 57.5 ± 11 nm were observed at residence times of 10 min and 12 min for 30 wt% and 35 wt% monomer concentrations, respectively.

Figure 4c–f display FE-SEM images illustrating the morphology of dried polystyrene particles synthesized in the AFR under varying conditions. Both AFR-S20% (Figure 4c,f) and AFR-S30% (Figure 4d,g) yielded uniform, well-defined, and individually dispersed polystyrene spheres with diameters around ~50 nm. In contrast, AFR-S35% (Figure 4e,h) also produced separated spheres; however, a clear size heterogeneity was observed, with some particles reaching 80–100 nm. This variation in the particle size may result from reduced latex stability at higher monomer concentrations or lower total flow rates during polymerization.

##### Continuous Operation and Fouling Analysis

**Figure 5:**
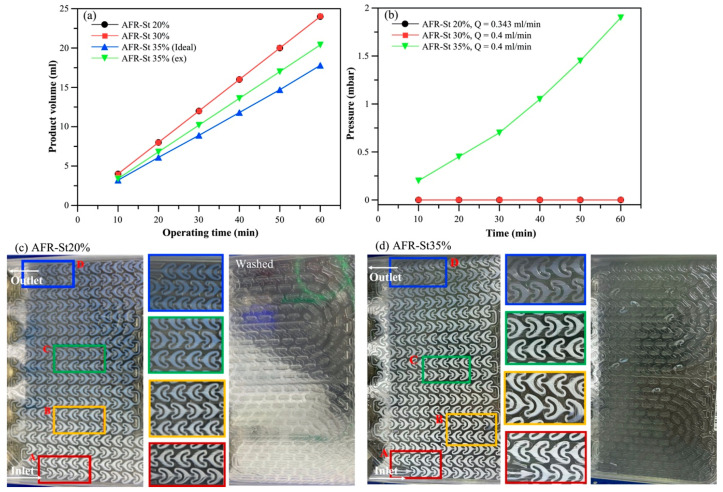
Performance of styrene emulsion polymerization in the AFR at different monomer concentrations. (**a**) Operational stability; (**b**) system pressure; (**c**) images of AFR-S20% during the experiment and after washing with deionized water; (**d**) images of AFR-S35% during the experiment and after washing, letters A–D show magnified images for different areas from the inlet to the outlet.

To evaluate the operational stability of the AFR system, a graduated cylinder was positioned at the reactor outlet to measure the volume of product collected over 60 min of continuous operation. Simultaneously, the internal pressure was monitored using pressure sensors installed at both the inlet and outlet of the AFR system. As shown in Figure 5a, the AFR continuously operated at a total flow rate of 0.4 mL/min for 60 min. During this period, approximately 4 mL of product was collected every 10 min, indicating stable polymerization without clogging at monomer concentrations of 20 wt% and 30 wt%. However, slight clogging occurred at AFR-S35%, resulting in less than 4 mL of product collected every 10 min. Additionally, the system pressure was observed to increase progressively with the operating time, rising from 0.2 mbar at 10 min to 1.9 mbar at 60 min. The pressure variation over time is illustrated in Figure 5b.

Enlarged images of each section of the AFR during polymerization are shown in Figure 5c,d. It can be observed that, during polymerization with a monomer concentration of 20 wt% and a total flow rate of 0.2 mL/min, no polystyrene formation was detected in the reaction module of the AFR. After completing polymerization with 20 or 30 wt% monomer concentrations, the AFR can be effectively cleaned within 30 min using deionized water at a total flow rate of 5 mL/min, without requiring any organic solvents. However, at a monomer concentration of 35 wt% (Figure 5b), clogging was observed when operating at a total flow rate of 0.229 mL/min, accompanied by the formation of milky white flocculent precipitates within the reaction module. Effective cleaning of the AFR under these conditions required flushing with acetone at a flow rate of 5 mL/min.

##### Comparison of Three Reactor Systems

Notably, the AFR exhibits superior mixing performance at a total flow rate close to 3 mL/min [20]. However, in the absence of an external pipeline, extending the residence time requires reducing the flow rate due to equipment constraints. This reduction in the total flow rate significantly diminishes the AFR’s mixing efficiency. In polymerization systems, the lower flow rate can also increase the likelihood of monomer adhesion to the reactor walls, potentially leading to clogging.

**Figure 6:**
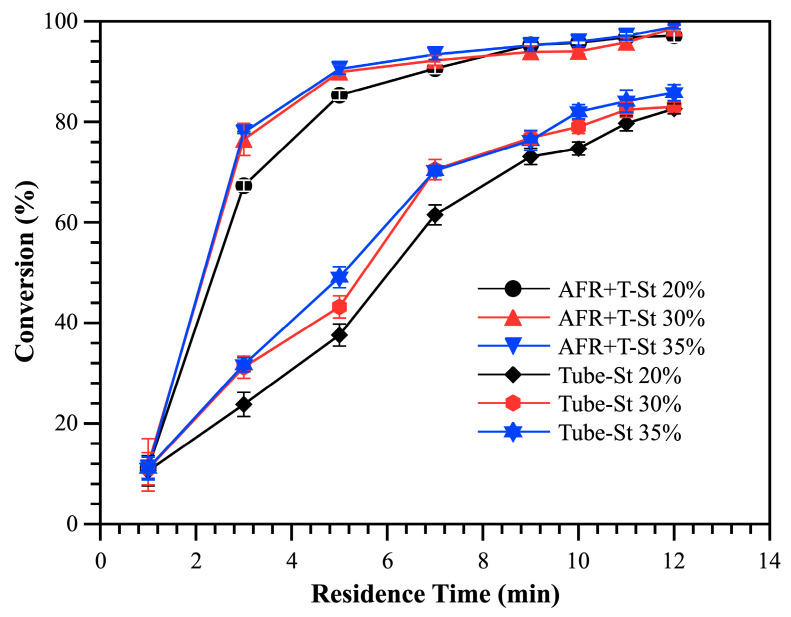
Comparison of styrene conversion across different reactor systems.

To overcome the limitations of the monolithic AFR and extend the residence time of the polymerization reaction, a straight PTFE pipe was connected to the AFR outlet. As shown in Figure 6, the addition of this external pipe effectively enhances styrene conversion across various monomer concentrations, even at a total flow rate of 2.6 mL/min. For example, at a residence time of 5 min and a monomer concentration of 20 wt%, the styrene conversion reached 85.29%. To achieve a comparable conversion using a single AFR, a much longer residence time of 13.65 min would be required, necessitating a reduced total flow rate of 0.2 mL/min.

Furthermore, in the AFR system connected with an external straight tube, the styrene conversion gradually plateaus after an additional 5 min of residence time, indicating that the polymerization rate approaches a steady state. At a maximum residence time of approximately 12 min, the polymerization conversion rates for all three monomer concentrations approached nearly 98%, demonstrating efficient monomer utilization under these conditions.

In contrast, a homemade tubular reactor, consisting of a simple 1/8-inch PTFE straight pipe, was constructed to compare the polymerization efficiency against that of the AFR external pipe system. This tubular reactor depends exclusively on a single T-shaped pipe fitting for mixing, with its length and experimental parameters set to regulate the residence time. As illustrated in Figure 6, the final conversion rates for Tube-St at monomer concentrations of 20, 30, and 35 wt% remained consistently low, reaching only about 82%.

Analyzing the polymerization conversion trends reveals that the AFR system connected to a straight tube delivers the best performance. This enhanced efficiency is likely attributed to the AFR’s microstructured design, which ensures significantly higher mixing efficiency under high-flow-rate conditions. This accelerated mixing promotes the diffusion of the initiator into monomer microcells, while the extended reaction time provided by the external straight tubes further enhances the overall conversion. When examining the molecular weight and molecular weight distribution (Đ) at a fixed residence time of approximately 10 min across various monomer concentrations and reactor systems, distinct differences become apparent.

In the single (monolithic) AFR, equipment limitations necessitate a reduced flow rate to achieve a longer residence time. However, this reduction compromises the mixing efficiency, resulting in a relatively broad polystyrene molecular weight distribution, with a Đ of approximately 1.66. Conversely, the external straight tube setup achieved a narrower molecular weight distribution (Đ = 1.47), even at high monomer concentrations. In contrast, the tubular reactor exhibited significantly broader molecular weight distributions, with Đ values around 2.5, due to its poor mixing efficiency—where the initiator and monomer are mixed only once.

### 3.3. AFR Used in Co-Emulsion Polymerization

**Figure 7:**
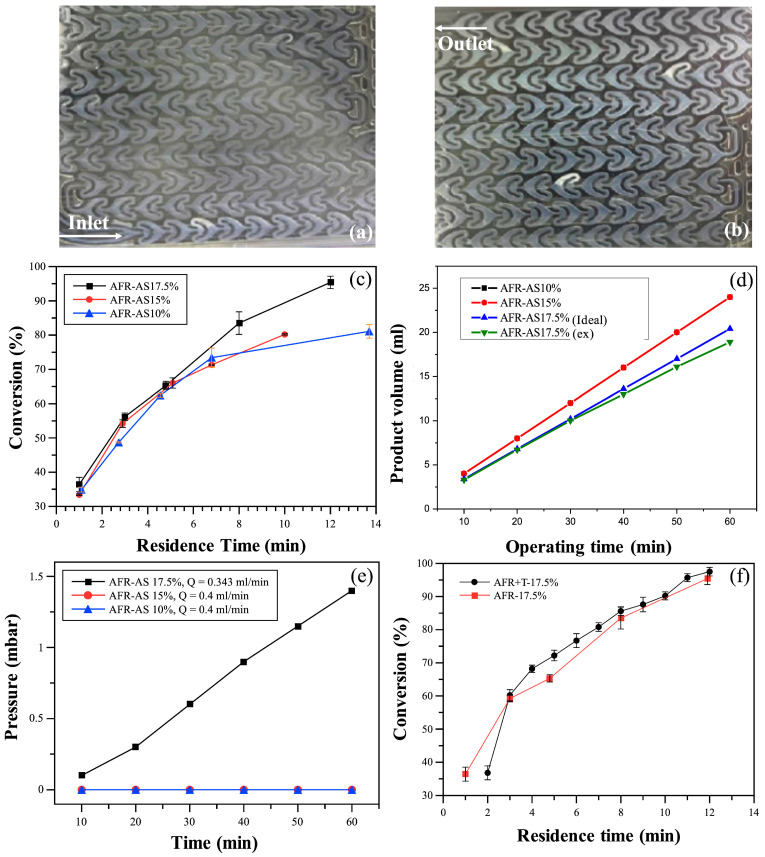
(**a**) Image of the inlet and (**b**) outlet of the AFR-AS 17.5% experiment at a total flow rate of 0.229 mL/min; (**c**) effect of residence time on conversion; (**d**) working stability of AFR; (**e**) system pressure changes in AFR for styrene acrylic emulsion copolymerization with different monomer concentrations; and (**f**) performance of the AFR system connected to a straight tube at a relatively high total flow rate (~2.7 mL/min).

Further investigation into the operability of the AFR was conducted using the synthesis of styrene–acrylic acid (St/AA) latex. As shown in Figure 7, polymerization of the St/AA co-emulsion at a 1:1 molar ratio exhibited similar trends across different monomer concentrations.

#### 3.3.1. Clogging in Single AFR Operation

Despite achieving a high conversion rate, clogging was observed in the microchannels of the AFR’s reaction module, as shown in Figure 7a,b. Among the tested conditions (Figure 7c), the AFR-AS17.5% demonstrated the highest polymerization rate, achieving a conversion of 95.42% at a residence time of approximately 12 min. The resulting latex had an average particle size of 288.5 ± 7 nm.

AFR-AS15% showed the second-best performance, achieving a conversion of 80.16% at a residence time of 10 min, with a corresponding latex particle size of 69.50 ± 7 nm. In contrast, AFR-AS10%, despite having the longest residence time of 14 min, resulted in a slightly higher conversion of 81.05%, but produced significantly smaller latex particles, with a size of 31.1 ± 0.2 nm.

The performance after 60 min of continuous operation is summarized in Figure 7d. The outlet product volume for AFR-AS17.5% (experimental data) exhibited a more gradual slope compared to the theoretical no-clogging scenario, with less than 2% of the total product volume collected every 10 min. Concurrently, the system pressure (Figure 7e) increased steadily from 0.1 mbar at 10 min to 1.4 mbar at 60 min.

Although a significant increase in the latex particle size was observed during polymerization in the AFR system compared to the initial droplet size of the St/AA emulsion, this growth mechanism alone cannot fully account for the substantial fouling observed in systems with 10 wt% and 17.5 wt% St/AA monomer concentrations. A more plausible explanation is the occurrence of particle aggregation during these experiments, promoted by two key factors: (i) the use of relatively high concentrations of the moderately water-soluble monomer acrylic acid (AA), and (ii) the application of relatively low total flow rates. Acrylic acid (AA) also functions as a co-surfactant alongside SDS, helping to form micelles that arrange in the outer layer of monomer droplets [37,38]. During nucleation, polymerization can destabilize these micelles because the formation of latex particles demands more surfactant to maintain stability [34,36]. However, as AA is consumed to form the polymer, the available co-surfactant decreases. This leads to unstable newly formed particle precursors, which are then either captured by existing particles or deposited on the reactor walls, causing significant fouling [8,10].

#### 3.3.2. Effect of Increasing Total Flow Rate on AFR Performance

To further clarify the impact of the total flow rates, the performance of the AFR system connected to a straight tube was investigated at a relatively high total flow rate (~2.7 mL/min). As shown in Figure 7f, a conversion of 97.49% was achieved at a residence time of approximately 12 min, producing a polymer with Mn = 2394 kDa, Mw = 2734 kDa, and a Đ of 1.14.

Compared to the single AFR results—which achieved a 95.42% conversion at a 12 min residence time for the same monomer concentration (with Mn = 3553 kDa, Mw = 4587 kDa, and Đ = 1.3)—the increase in the total flow rate did not lead to a notable improvement in conversion rates or a significant reduction in fouling. Nonetheless, the higher total flow rates clearly improved the molecular weight distribution, yielding a narrower Đ.

## 4. Conclusions

This study aimed to evaluate the practical applicability of three continuous-flow reactors—a homemade tube reactor, the monolithic Corning Advanced-Flow Reactor (AFR), and the AFR coupled with an external PTFE pipe—for emulsion polymerization. The evaluation focused on key performance metrics including monomer conversion, operational stability, pressure drop, and internal reactor imaging.

The monolithic AFR demonstrated high monomer conversions; however, it was susceptible to fouling and clogging at elevated monomer concentrations, especially when the flow rates were reduced to increase the residence time. This issue was mainly due to diminished mixing efficiency and enhanced particle aggregation within the reactor.

The homemade tube reactor consistently exhibited lower monomer conversions and broader molecular weight distributions, primarily due to its limited mixing efficiency. In contrast, the AFR coupled with an external PTFE pipe proved to be the most effective system, offering enhanced performance and improved control over polymer characteristics. This setup combined the AFR’s superior mixing at high flow rates with the flexibility to independently extend the residence time, resulting in the highest conversions (approaching 98% for St), reduced fouling, and a consistently narrower molecular weight distribution (Đ~1.14–1.47).

This work highlights the key importance of the reactor design and flow dynamics in continuous emulsion polymerization. Our findings support the use of hybrid reactor configurations, such as the AFR combined with an external pipe, as a robust and efficient solution for polymer emulsion synthesis. This approach offers improved process control and scalability, making it highly promising for a wide range of industrial applications. 

Based on our findings, future research will aim to optimize the hybrid reactor system for long-term industrial applications. This will include a detailed investigation of internal reaction surface contamination, providing essential data to validate the long-term operational stability and antifouling performance of the AFR-pipe hybrid.

## Data Availability

The original contributions presented in this study are included in the article/Supplementary Material. Further inquiries can be directed to the corresponding author.

## Supplementary Materials

The following supporting information can be downloaded at: https://www.mdpi.com/article/10.3390/polym17172289/s1; Table S1: feed formulations; Table S2: operating parameters.
