# Supplementary material for: Comparative Study of Continuous-Flow Reactors for Emulsion Polymerization
Source: Polymers (Basel). 2025 Aug 24;17(17):2289. doi: 10.3390/polym17172289 (PMC12430675; doi:10.3390/polym17172289)
Supplement: Supplementary file 1 [file polymers-17-02289-s001.zip › polymers-3826349-supplementary.pdf]

Comparative Study of Continuous-Flow Reactors for Emulsion Polymerization

Kai-Yen Chin[1,†], Angus Shiue[1,†], Pei-Yu Lai[1], Shu-Mei Chang[1,*], Graham Leggett[2]

[1] Department of Molecular Science and Engineering, National Taipei University of Technology, Taipei, Taiwan; f104gstyle@gmail.com (K.-Y.C.); angusshiue@gmail.com (A.S.); knight32671@gmail.com (P.-W.L.)
[2] LI-COR, Lincoln, Nebraska, USA; graham.leggett@licor.co
* Correspondence: f10914@mail.ntut.edu.tw (S.M.C)
† Denotes equal contribution.

Supporting Information

Table S1. Feed formulations

**Code**			**Feed ratio**		**Monomer C* in reactor (wt%)**
			Feed A*	Feed B	
**S20%**			1	1	20
**S30%**			3	1	30
**S35%**			7	1	35
	AA/St Molar ratios				
**AS10%**	1	1	1	1	10
**AS15%**	1	1	3	1	15
**AS17.5%**	1	1	7	1	17.5

C*: Concentration, Feed A*: 40wt% St emulsion or 20 wt% AA/St emulsion.

Table S2. Operating parameters

AFR								
St20% or AS10%			St30% or AS15%			St35% or AS17.5%		
Feed A (ml/min)	Feed B (ml/min)	RT (min)	Feed A (ml/min)	Feed B (ml/min)	RT (min)	Feed A (ml/min)	Feed B (ml/min)	RT (min)
0.1	0.1	13.7	0.2	0.07	10	0.2	0.03	12
0.2	0.2	6.8	0.3	0.1	6.8	0.3	0.04	8
0.3	0.3	4.6	0.4	0.13	5.1	0.5	0.07	4.8
0.5	0.5	2.7	0.7	0.23	2.9	0.8	0.12	3
1.3	1.3	1.1	2	0.67	1	2.3	0.33	1

AFR connected with external pipe										
		St20% or AS10%			St30% or AS15%			St35% or AS17.5%		
External pipe length (m)	AFR+Pipe inner volume (ml)	Feed A (ml/min)	Feed B (ml/min)	RT (min)	Feed A (ml/min)	Feed B (ml/min)	RT (min)	Feed A (ml/min)	Feed B (ml/min)	RT (min)
1.25	5.24	1.3	1.3	2	2	0.67	2	2.3	0.33	2
2.5	7.76	1.3	1.3	3	2	0.67	3	2.3	0.33	3
3.75	10.27	1.3	1.3	4	2	0.67	3.9	2.3	0.33	3.9
5	12.78	1.3	1.3	4.9	2	0.67	4.8	2.3	0.33	4.9
6.25	15.29	1.3	1.3	5.9	2	0.67	5.7	2.3	0.33	5.8
7.25	17.8	1.3	1.3	6.9	2	0.67	6.7	2.3	0.33	6.8
8.75	20.31	1.3	1.3	7.8	2	0.67	7.6	2.3	0.33	7.7
10	22.82	1.3	1.3	8.8	2	0.67	8.6	2.3	0.33	8.7

11.25	25.33	1.3	1.3	9.8	2	0.67	9.5	2.3	0.33	9.6
12.5	27.84	1.3	1.3	10.7	2	0.67	10.4	2.3	0.33	10.6
13.75	30.35	1.3	1.3	11.7	2	0.67	11.4	2.3	0.33	11.6

Tubular reactor

		T-S20%			T-S30%			T-S35%		
External pipe length (m)	AFR+Pipe inner volume (ml)	Feed A (ml/min)	Feed B (ml/min)	RT (min)	Feed A (ml/min)	Feed B (ml/min)	RT (min)	Feed A (ml/min)	Feed B (ml/min)	RT (min)
1.35	2.71	1.3	1.3	1	2	0.67	1	2.3	0.33	1
2.6	5.22	1.3	1.3	2	2	0.67	2	2.3	0.33	2
3.85	7.73	1.3	1.3	3	2	0.67	2.9	2.3	0.33	2.9
5.1	10.24	1.3	1.3	3.9	2	0.67	3.8	2.3	0.33	3.9
6.35	12.75	1.3	1.3	4.9	2	0.67	4.8	2.3	0.33	4.9
7.6	15.26	1.3	1.3	5.9	2	0.67	5.7	2.3	0.33	5.8
8.85	17.77	1.3	1.3	6.8	2	0.67	6.7	2.3	0.33	6.8
10.1	20.28	1.3	1.3	7.8	2	0.67	7.6	2.3	0.33	7.72\
11.35	22.79	1.3	1.3	8.8	2	0.67	8.6	2.3	0.33	8.7
12.6	25.30	1.3	1.3	9.7	2	0.67	9.5	2.3	0.33	9.6
13.85	27.81	1.3	1.3	10.7	2	0.67	10.4	2.3	0.33	10.6
15	30.13	1.3	1.3	11.6	2	0.67	11.3	2.3	0.33	11.5
